# Utilization of the GOAL questionnaire as a standardized screening tool for obstructive sleep apnea

**DOI:** 10.1038/s41598-023-31247-x

**Published:** 2023-04-04

**Authors:** Yanqing Xing, Zhenxia Zhang, Jiansheng Yin, Yi Liu, Ziwei Shuai, Zhihong Liu, Xinrui Tian, Shouan Ren

**Affiliations:** 1 Department of Respiratory and Critical Care Medicine, The Second Hospital of Shanxi Medical University, No. 382, Wuyi Road, Xinghualing District, Taiyuan, Shanxi China; 2 Department of Respiratory and Critical Care Medicine, The First Hospital of Shanxi Medical University, No. 85, Jiefang South Road, Yingze District, Taiyuan, Shanxi China

**Keywords:** Diseases, Health care, Medical research

## Abstract

The purpose of our study was to evaluate the application value of the GOAL questionnaire in screening obstructive sleep apnea (OSA) and to compare it with the other three questionnaires in sleep clinics. A cross-sectional study was conducted in 436 patients who had undergone nocturnal polysomnography in the sleep unit of the First Hospital of Shanxi Medical University between September 2021 and May 2022, and all patients completed the four questionnaires (GOAL questionnaire, STOP-Bang questionnaire, NoSAS score and No-Apnea score) truthfully, and the patients were divided into 3 groups: AHI ≥ 5 events/h group, AHI ≥ 15 events/h group and AHI ≥ 30 events/h group. The predictive effect of the questionnaire on different AHI cut-off values was calculated, and performance of four questionnaires was assessed by the discriminatory ability. This study ultimately included 410 patients, and there were statistically significant differences in gender, age, BMI, neck circumference, clinical symptoms, hypertension, diabetes, AHI, and minimum oxygen saturation between OSA and non-OSA groups (P < 0.05). The AUC for No-Apnea score was 0.79, the AUC for STOP-Bang questionnaire was 0.86, the AUC for NoSAS score was 0.81, and the AUC for GOAL questionnaire was 0.77. These four questionnaires were effective in screening OSA when AHI ≥ 15 events/h. Similar to No-Apnea score, STOP-Bang score and NoSAS score, GOAL questionnaire has a good predictive value for OSA, which is a questionnaire suitable for primary health-care centers and clinics.

## Introduction

Obstructive sleep apnea is a highly prevalent sleep-related respiratory disorder^1^, affecting at least 2% of adult women and 4% of adult men worldwide^2^, and recent estimates suggested that nearly 1 billion people might be affected, with approximately 425 million OSA patients worldwide between the ages of 30 and 69 years and predominantly of moderate to severe severity^3^. OSA is characterized by repeated narrowing and closure of the upper airway during sleep, resulting in transitory respiratory arrests of at least 10 s or a significant reduction in tidal volume, which in turn causes intermittent hypoxia and sympathetic activation^4^, with frequent micro-awakening. The main symptoms are nocturnal snoring and daytime sleepiness, with apnea being the most specific manifestation.

OSA is a danger factor for hemorrhagic and ischemic cerebrovascular disease^5^, hypertension^6^, arrhythmias^7^, ischemic heart disease and heart failure^8^, diabetes^9^, cognitive impairment^10^, and traffic accidents due to reduced alertness and attention^11^. Therefore, early diagnosis of OSA is important. Nocturnal polysomnography (PSG) is the gold standard for the diagnosis of OSA, but it is expensive and time consuming, requiring trained personnel and advanced equipment. Limited availability of PSG requires the establishment of alternative methods for diagnosing patients with moderate to severe OSA. Simple and reliable screening tools are needed to screen patients with high-risk OSA and stratify them for definitive PSG diagnosis or further treatment.

There are now a large number of clinical alternatives that can screen OSA. And these are broadly divided into clinical prediction models^12,13^ and questionnaires. Clinical models designed to screen OSA require specific techniques such as cephalometric, morphometric measurements and the assistance of a computer. Although the test accuracy is high, these models are not appropriate for clinical practice. In recent years, several OSA screening tools based on questionnaires have been developed. The ideal OSA screening tool should have high sensitivity to reduce the rate of missed diagnosis and certain specificity to prevent overdiagnosis. These screening questionnaires such as Berlin questionnaire, Epworth Sleepiness Scale, No-Apnea score, STOP-Bang questionnaire, NoSAS score and GOAL questionnaire. The Berlin Questionnaire was developed in the primary health-care population and is generally used in epidemiological and clinical studies. It is based on snoring, daytime sleepiness and metabolic disorders. Its disadvantages are more questions, more complexity, low patient completion and low specificity. The Epworth Sleepiness Scale, which assesses daytime sleepiness, is a tool for determining subjective sleepiness. Its drawbacks are that the information is derived from patients, the risk is underestimated, and evaluation ability is limited because the severity of OSA is not always positively correlated with daytime sleepiness. The No-Apnea score consists of objective information, reducing subjective bias. Its disadvantage is that the scoring mechanism is complicated. If the age of the patient is greater than or equal to 55 years old, he will directly get 3 points, which will cause the patients aged 55 years and above to directly become the high-risk group, resulting in a certain bias. The STOP-Bang questionnaire is simple, quick, easy to use, highly sensitive and widely used in surgical patients, but it includes 3 subjective variables and has limited detectability in patients undergoing bariatric surgery and in pregnant women who are predominantly young^14,15^. The NoSAS score has more objective biometric items and fewer subjective variables; it has the disadvantage that each variable is assigned a different score and the scoring mechanism is more complex. The GOAL questionnaire, which includes both subjective and objective variables, is the simplest volume. It is a newly developed questionnaire that has not been widely verified.

The GOAL questionnaire is a newly developed and validated practical questionnaire by Ricardo LM Duarte^16^, which includes both subjective and objective variables and provides yes–no dichotomous answers. The GOAL questionnaire includes gender, obesity, age and loud snoring, with items scoring 0–4, with a score greater than or equal to 2 indicating a higher risk of OSA. This focuses on prioritizing sensitivity over specificity and aims to reduce the false negative rate. The GOAL questionnaire is currently less applied, therefore, in this study we aimed to validate the application value of the GOAL questionnaire in screening OSA and compare it with the other three questionnaires (No-Apnea score, STOP-Bang questionnaire and NoSAS score).

## Methods

### Object of the study

Four hundred and thirty-six patients who visited the sleep unit of the first Hospital of Shanxi Medical University between September 2021 and May 2022 were selected. Inclusion criteria: age > 18 years, independent behavioral and cognitive abilities, total sleep time > 4 h, ability to respond to questionnaires completely and complete anthropometric data. Exclusion criteria: history of brain tumor or epilepsy, benzodiazepine use, sleep disorders other than OSA, patients with neuromuscular diseases or other pulmonary diseases affecting blood oxygenation, patients treated with CPAP (continuous positive airway pressure ventilation), refusal to monitor, immature polysomnography or previously diagnosed as OSA. The study protocol was in strict accordance with the Declaration of Helsinki in 1964 and was previously approved by the Ethics Committee of the first Hospital of Shanxi Medical University (number2021-K117). Written informed consent was obtained from all volunteers, and the anonymity of each participant was strictly preserved.

### Data collection

The sleep technician measured and documented the patient's age, gender, height, weight, neck circumference, blood pressure, and other measurements. BMI (Body mass index) was calculated by dividing weight (in kilograms) by the square of height (in meters), while NC (neck circumference) was measured systematically with a flexible tape measure, with all subjects in an upright sitting position and the upper edge of the tape measure placed directly below the throat bulge and perpendicular to the long axis of the neck. All subjects completed the No-Apnea score, STOP-Bang questionnaire, NoSAS score and GOAL questionnaire. Questionnaire scores were calculated on the bases of anthropometric and demographic data collected. The PSG was administered that night and data recorded during the patient's sleep included snoring and apnea, as well as their severity and duration.

### Screening tools

STOP-Bang questionnaire^17^ is presented in Table S1: The STOP-Bang questionnaire consists of eight yes or no questions: loud snoring, daytime fatigue, apnea observed during sleep, hypertension, BMI > 30 kg/m^2^, age > 50 years, neck circumference > 40 cm and male. A “yes” answer was scored as 1 and a “no” answer was scored as 0. The risk of OSA was higher if the score was greater than or equal to 3.

NoSAS score^18^ is presented in Table S2: A score of 4 is given for NC greater than 40 cm, 3 for BMI greater than 25 kg/m^2^, 5 for BMI greater than or equal to 30 kg/m^2^, 2 for snoring, 4 for aged 55 years or older, and 2 for men. The NoSAS score ranges from 0 to 17. A NoSAS score of 8 or higher is considered to be at high-risk for OSA.

No-Apnea score^19^ is presented in Table S3: the No-Apnea score includes NC and age, with NC scored as 37.0–39.9 cm (1 point), 40.0–42.9 cm (3 points), and ≥ 43.0 cm (6 points); and age scored as 35–44 years (1 point), 45–54 years (2 points), and ≥ 55 years old (3 points). The points for each variable were summed for a final score of 0–9. A score greater than or equal to 3 was thought to be a high-risk for OSA.

GOAL questionnaire^16^ is presented in Table S4: The GOAL questionnaire is a newly developed questionnaire that contains both subjective and objective variables. The GOAL questionnaire contains gender, obesity, age and loud snoring, with items scoring 0–4. A score greater than or equal to 2 was considered to be a high-risk for OSA.

### PSG monitoring

We monitored patients for at least 7 h using Alice 5 PSG (Philips) portable testing device. The signal recording included airflow from the nasal pressure sensor, peripheral oxygen saturation, snoring, chest and abdominal movements via the respiratory sensing volume tracing belt, and body position. All sleep recordings were analyzed by sleep professionals according to relevant guidelines and subsequently reviewed by a sleep physician, according to the AASM (American Academy of Sleep Medicine) standards^20^, being that both physicians were blind to the four scores, which were collected prior to PSG. Referring to 2017 AASM Scoring Manual Version 2.4 on the interpretation of sleep and related events^21^, apnea was defined as ≥ 90% reduction in airflow for at least 10 s, and hypoventilation was defined as ≥ 30% reduction in airflow for at least 10 s and ≥ 3% decrease in oxygen saturation.

### Statistical analysis

This study summarizes demographic, sleep studies and screening questionnaire data and uses SPSS 21.0 (SPSS Inc, Chicago, USA) for statistical analysis. Normally distributed data were expressed as mean and standard deviation, and qualitative variables were presented as frequency (N) and percentages (%). Comparisons between groups were tested by χ test and t-test. P < 0.05 was defined as statistically significant. To assess the performance of the four questionnaires in screening OSA, sensitivity, specificity, positive predictive values (PPVs) and negative predictive values (NPVs) were calculated for four questionnaires and at different AHI cut-offs. Area under the receiver-operating curve (ROC) was also calculated to assess the diagnostic ability at different AHI cut-offs.

### Ethical approval

All procedures performed in studies involving human participants were in accordance with the ethical standards of the Ethics Committee of the first Hospital of Shanxi Medical University (number 2021-K117) and the 1964 Helsinki declaration and its later amendments or comparable ethical standards.

### Informed consent

Informed consent was obtained from all individual participants included in the study.

## Results

### Demographic data and sleep characteristics

**Figure 1:**
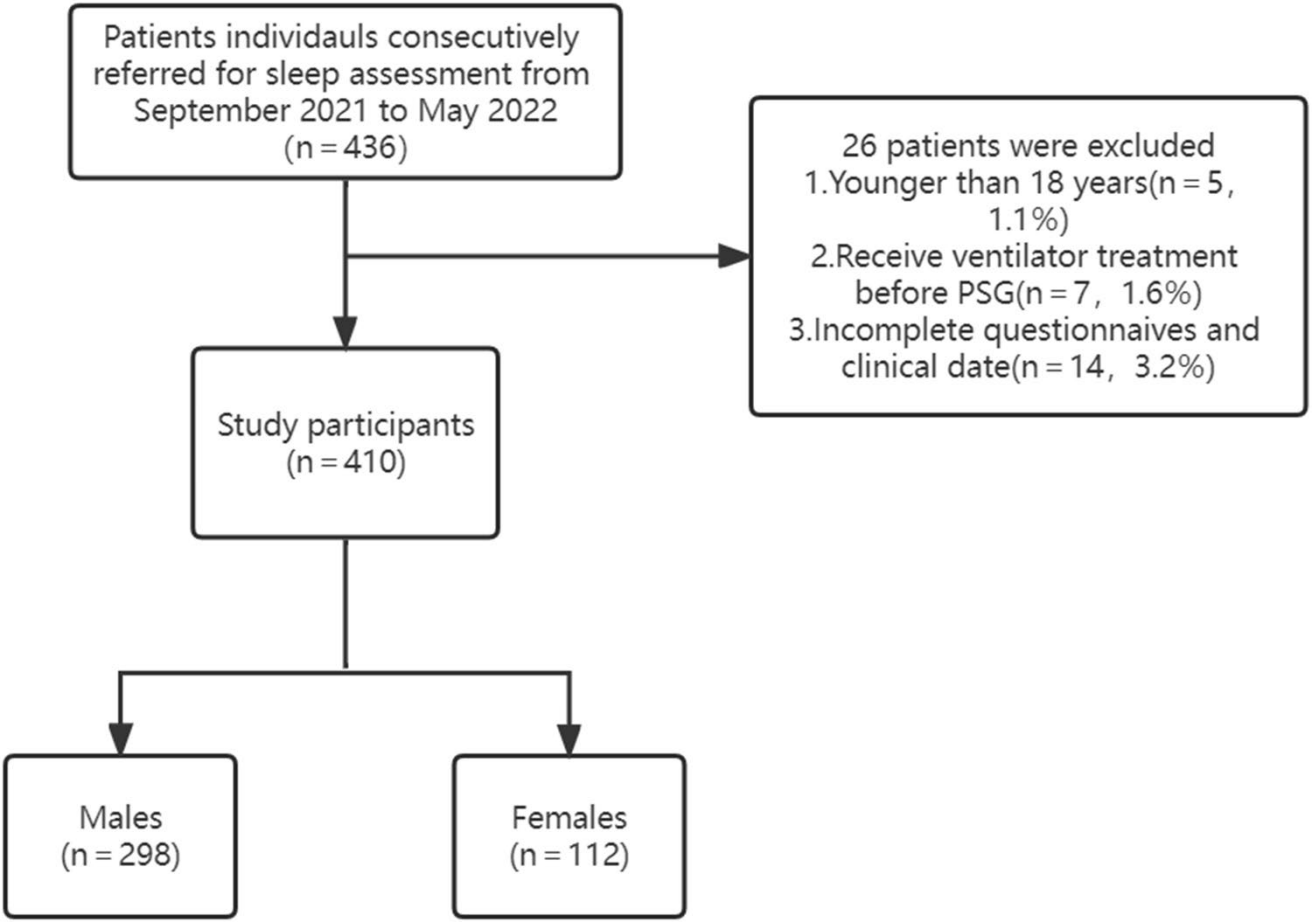
Flow chart showing the subject enrollment. *PSG* polysomnography.

From 436 patients who visited the sleep laboratory, 410 patients were finally included in our study according to the inclusion and exclusion criteria, as can be seen in Fig. 1.

The demographic and sleep study results of the studied subjects are presented in Table 1. We observed that compared with the non-OSA group, the OSA group showed significant differences (P < 0.05) in gender, age, BMI, neck circumference, hypertension, diabetes, loud snoring, observed apnea, tiredness et al.

**Table 1:** Demographic and polysomnographic characteristics (n = 410).

Parameter	Total number of patients (n = 410)	Simple snoring group (n = 89)	OSA group (n = 321)	P-value
Male (n [%])^a^	298 (72.7)	41 (46.07)	257 (80.06)	< 0.001*
Age (year)^b^	45.59 ± 14.30	39.7 ± 14.12	47.54 ± 13.69	0.001*
BMI (kg/m^2^)^b^	27.91 ± 4.47	24.9 ± 4.07	28.70 ± 4.25	< 0.001*
NC (cm)^b^	38.97 ± 4.12	35.44 ± 3.04	39.90 ± 3.89	< 0.001*
Hypertension (n [%])^a^	207 (50.5)	24 (26.97)	183 (57.01)	< 0.001*
Diabetes (n [%])^a^	108 (26.3)	12 (13.5)	96 (29.9)	0.002*
Coronary disease (n [%])^a^	52 (12.7)	9 (10.1)	43 (13.4)	0.278
CVD (n [%])^a^	20 (4.9)	4 (4.5)	16 (5.0)	0.849
Loud snoring (n [%])^a^	347 (84.6)	32 (35.96)	315 (98.13)	< 0.001*
Observed apnea (n [%])^a^	200 (48.7)	7 (7.87)	193 (60.12)	< 0.001*
Tiredness (n [%])^a^	228 (55.6)	20 (22.47)	208 (64.80)	< 0.001*
No-Apnea(points)^b^	3.75 ± 2.29	1.61 ± 1.43	4.35 ± 2.13	< 0.001*
STOP-Bang(points)^b^	4.04 ± 1.77	1.80 ± 1.05	4.70 ± 1.38	< 0.001*
NoSAS (points)^b^	8.90 ± 4.13	3.99 ± 2.69	10.27 ± 3.44	< 0.001*
GOAL (points)^b^	2.25 ± 0.88	1.21 ± 0.66	2.56 ± 0.71	< 0.001*
ADA (s)^b^	23.01 ± 8.07	17.31 ± 8.80	24.50 ± 6.84	< 0.001*
MDA (s)^b^	51.36 ± 22.84	29.04 ± 22.87	57.61 ± 18.57	< 0.001*
ODI (n/h)^b^	30.34 ± 25.58	4.04 ± 3.41	37.35 ± 23.78	< 0.001*
NMSBP (mmHg)^b^	174.99 ± 31.72	156.52 ± 20.74	180.74 ± 32.00	< 0.001*
NMDBP (mmHg)^b^	94.25 ± 12.69	88.31 ± 8.443	96.04 ± 13.20	< 0.001*
AHI (events/h)^b^	32.30 ± 27.63	2.69 ± 1.14	40.69 ± 25.83	< 0.001*
Mean SpO_2_ (%)^b^	91.93 ± 4.65	94.36 ± 3.83	91.43 ± 4.42	< 0.001*
Minimum SpO_2_ (%)^b^	77.44 ± 11.93	86.79 ± 8.49	75.11 ± 11.29	< 0.001*

Comparisons between groups were tested by χ test and t-test. P < 0.05 was defined as statistically significant.

^a^Frequency (%) and χ test; ^b^Mean and variance, and t-test. *P < 0.05.

*BMI* body mass index, *NC* neck circumference, *CVD* cerebrovascular disease, *ADA* average duration of apnea, *MDA* maximum duration of apnea, *ODI* oxygen desaturation index, *NMSBP* nocturnal maximum systolic blood pressure, *NMDBP* nocturnal maximum diastolic blood pressure, *AHI* apnea hypopnea index, *Mean SpO*_*2*_ mean oxygen saturation, *Minimum SpO*_*2*_ minimum oxygen saturation.

Numeric and categorical variables were reported as mean ± standard deviation and N (%), respectively.

### Sensitivity and specificity of the four screening tools

The predictive performance of the four screening questionnaires is shown in Tables 2, 3 and 4. The sensitivity and specificity of the No-Apnea score were 84.42% and 68.54%, 87.02% and 52.03%, 88.52% and 39.65% when the AHI was ≥ 5, ≥ 15 and ≥ 30 events/h, respectively; the STOP-Bang score was 94.70% and 84.27%, 96.18% and 55.41%, 98.36% and 39.21%, respectively; the NoSAS score were 74.77% and 92.13%, 77.10% and 69.60%, 81.97% and 57.27%, respectively; the GOAL score were 95.95% and 77.53%, 96.95% and 50.00%, 98.36% and 34.80%, respectively. The GOAL questionnaire and STOP-Bang questionnaire had the highest sensitivity for screening OSA, followed by the No-Apnea score and NoSAS score, while the NoSAS score had the highest specificity for screening OSA.

**Table 2:** Predictive parameters of the four screening tools at AHI ≥ 5 events/h.

	No-Apnea	STOP-Bang	NoSAS	GOAL
Sensitivity	0.84 (0.80–0.88)	0.95 (0.91–0.97)	0.75 (0.70–0.79)	0.96 (0.93–0.98)
Specificity	0.69 (0.58–0.78)	0.84 (0.75–0.91)	0.92 (0.84–0.97)	0.78 (0.67–0.85)
PPV	0.91 (0.87–0.94)	0.96 (0.93–0.97)	0.97 (0.94–0.99)	0.94 (0.91–0.96)
NPV	0.55 (0.45–0.64)	0.82 (0.72–0.89)	0.50 (0.42–0.58)	0.84 (0.74–0.91)
+ LR	2.68 (1.97–3.66)	6.02 (3.72–9.74)	9.51 (4.66–19.41)	4.27 (2.90–6.28)
− LR	0.23 (0.17–0.30)	0.06 (0.04–0.10)	0.27 (0.23–0.33)	0.05 (0.03–0.09)
AUC (95% CI)	0.86 (0.81–0.90)	0.94 (0.92–0.97)	0.91 (0.87–0.94)	0.90 (0.86–0.94)

*AHI* apnea hypopnea index, *PPV* positive predictive value, *NPV* negative predictive value, *+ LR* positive likelihood ratio, *− LR* negative likelihood ratio, *AUC* area under the curve.

Data are presented as values (95% confidence intervals).

**Table 3:** Predictive parameters of the four screening tools at AHI ≥ 15 events/h.

	No-Apnea	STOP-Bang	NoSAS	GOAL
Sensitivity	0.87 (0.82–0.91)	0.96 (0.93–0.98)	0.77 (0.71–0.82)	0.97 (0.94–0.99)
Specificity	0.52 (0.44–0.60)	0.55 (0.47–0.63)	0.70 (0.61–0.77)	0.50 (0.42–0.58)
PPV	0.76 (0.71–0.81)	0.79 (0.74–0.83)	0.82 (0.76–0.86)	0.77 (0.72–0.82)
NPV	0.69 (0.60–0.78)	0.89 (0.80–0.94)	0.63 (0.55–0.70)	0.90 (0.81–0.95)
+ LR	1.81 (1.52–2.16)	2.16 (1.80–2.59)	2.54 (1.97–3.26)	1.94 (1.65–2.28)
− LR	0.25 (0.18–0.35)	0.07 (0.04–0.13)	0.33 (0.26–0.41)	0.06 (0.03–0.12)
AUC (95% CI)	0.79 (0.74–0.83)	0.86 (0.82–0.90)	0.81 (0.76–0.85)	0.77 (0.72–0.82)

*AHI* apnea hypopnea index, *PPV* positive predictive value, *NPV* negative predictive value, *+ LR* positive likelihood ratio, *− LR* negative likelihood ratio, *AUC* area under the curve.

Data are presented as values (95% confidence intervals).

**Table 4:** Predictive parameters of the four screening tools at AHI ≥ 30 events/h.

	No-Apnea	STOP-Bang	NoSAS	GOAL
Sensitivity	0.89 (0.83–0.93)	0.98 (0.95–1.00)	0.82 (0.75–0.87)	0.98 (0.95–1.00)
Specificity	0.40 (0.33–0.46)	0.39 (0.33–0.46)	0.57 (0.51–0.64)	0.35 (0.29–0.41)
PPV	0.54 (0.48–0.60)	0.57 (0.51–0.62)	0.61 (0.54–0.67)	0.55 (0.49–0.60)
NPV	0.81 (0.72–0.88)	0.97 (0.90–1.00)	0.80 (0.73–0.85)	0.96 (0.89–0.99)
+ LR	1.47 (1.30–1.65)	1.62 (1.46–1.80)	1.92 (1.63–2.26)	1.51 (1.37–1.66)
− LR	0.29 (0.19–0.44)	0.04 (0.01–0.13)	0.31 (0.23–0.43)	0.05 (0.02–0.15)
AUC (95% CI)	0.73 (0.68–0.78)	0.80 (0.76–0.84)	0.77 (0.73–0.82)	0.71 (0.67–0.76)

*AHI* apnea hypopnea index, *PPV* positive predictive value, *NPV* negative predictive value, *+ LR* positive likelihood ratio, *− LR* negative likelihood ratio, *AUC* area under the curve.

Data are presented as values (95% confidence intervals).

### AUC of the area under the ROC curve for the four screening tools

**Figure 2:**
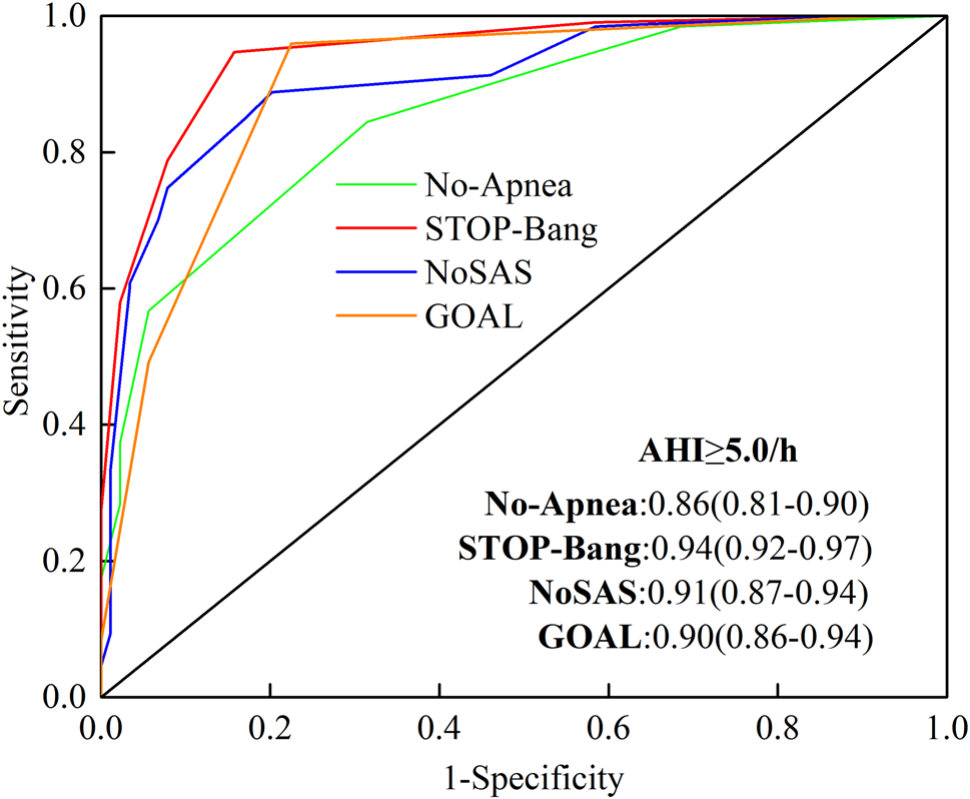
ROC curves of four screening tools (No-Apnea score, STOP-Bang questionnaire, NoSAS score and GOAL questionnaire) with an AHI cut-off ≥ 5 events/h. The area under the ROC curve (AUC) closer to 1 indicates the higher diagnostic accuracy of the screening questionnaire. AUC data are presented as values (95% confidence intervals). *ROC* receiver-operating curve, *AHI* apnea hypopnea index, *AUC* area under the curve.

**Figure 3:**
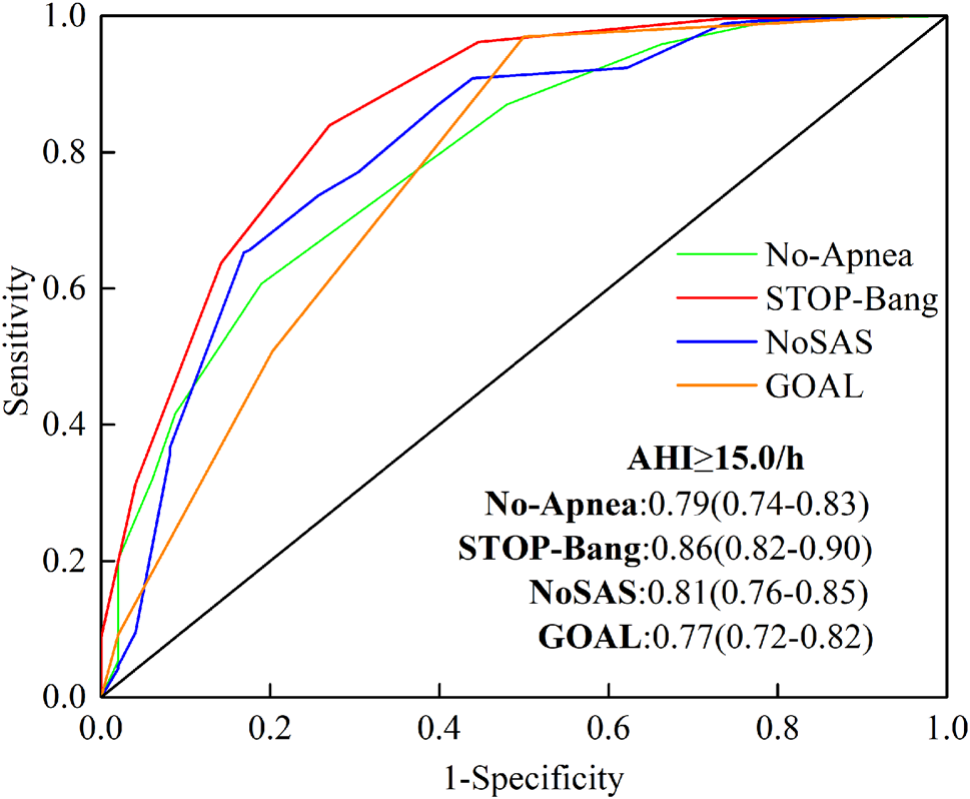
ROC curves of four screening tools (No-Apnea score, STOP-Bang questionnaire, NoSAS score and GOAL questionnaire) with an AHI cut-off ≥ 15 events/h. The area under the ROC curve (AUC) closer to 1 indicates the higher diagnostic accuracy of the screening questionnaire. AUC data are presented as values (95% confidence intervals). *ROC* receiver-operating curve, *AHI* apnea hypopnea index, *AUC* area under the curve.

**Figure 4:**
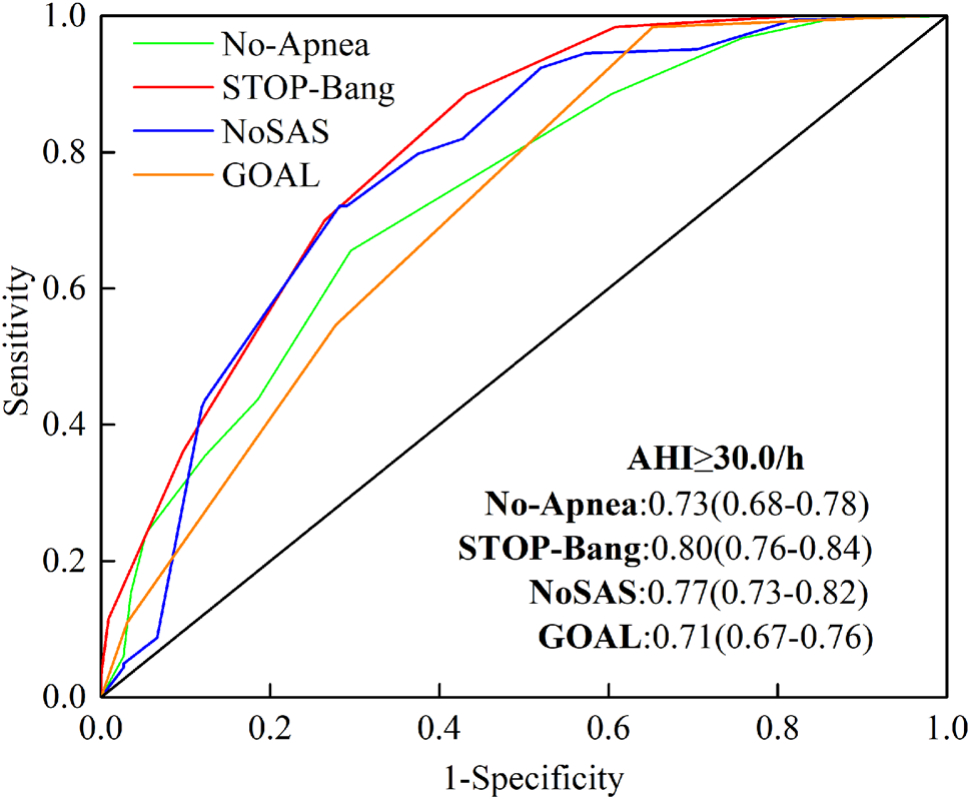
ROC curves of four screening tools (No-Apnea score, STOP-Bang questionnaire, NoSAS score and GOAL questionnaire) with an AHI cut-off ≥ 30 events/h. The area under the ROC curve (AUC) closer to 1 indicates the higher diagnostic accuracy of the screening questionnaire. AUC data are presented as values (95% confidence intervals). *ROC* receiver-operating curve, *AHI* apnea hypopnea index, *AUC* area under the curve.

As shown in Figs. 2, 3, and 4, the area under the curve (AUC) of the four questionnaire scores were compared using AHI 5, 15, and 30 events/h as the threshold values, showing that the AUC values for the No-Apnea score were 0.86, 0.79, and 0.73 for AHI ≥ 5, 15, and 30 events/h, respectively; the STOP-Bang were 0.95, 0.86, and 0.80, respectively; the NoSAS were 0.91, 0.81, and 0.77, respectively; and GOAL were 0.90, 0.77, and 0.71, respectively. STOP-Bang had the highest AUC values.

## Discussion

This study collected patients attending a sleep laboratory and the results showed that four questionnaires had good screening ability in screening patients at high-risk OSA. This study reveals that the GOAL questionnaire is not inferior to the No-Apnea score, STOP-Bang questionnaire and NoSAS score in discriminating the severity of OSA.

OSA is a highly prevalent and often underdiagnosed disease^1^^.^, multiple nocturnal hypoxia and abnormal sleep architecture caused by prolonged collapse or obstruction of the upper airway is responsible for OSA, culminating in some degree of damage to multiple target organs and a threat to the patient's quality of life. It is significantly related to coronary artery disease, hypertension, diabetes mellitus, and cerebrovascular^22^. OSA directly or indirectly increases health care and economic costs. PSG is the gold standard for the diagnosis of OSA. However, the prevalence of OSA is much higher than the number of patients that can be handled by existing sleep laboratories, therefore, considering the limited availability of PSG and the importance of screening for OSA in primary health-care hospitals, patients at high-risk of OSA can be identified first with a sleep apnea screening questionnaire, so that medical resources can be better utilized to patients at high-risk of OSA.

According to the study, these 410 patients belonged to a high prevalence group of OSA and were predominantly male. The prevalence of OSA is high in the elderly^23^, and the higher the age, the higher the prevalence of OSA increases, and our study also confirmed a statistically significant difference in the age of OSA patients compared with non-OSA patients (P < 0.05). Some studies have shown that women have a lower prevalence than men, but the prevalence increases significantly with age in postmenopausal women^24^, and the difference between the sexes is significantly smaller at this time^25^. Some studies have demonstrated that the clinical symptoms are different in male and female OSA patients. Typical clinical symptoms are nocturnal snoring and apnea in male OSA patients, while female OSA patients are usually atypical, such as insomnia, headache, and fatigue^26^. Some studies have demonstrated that in patients with OSA, the neck circumference of men is larger than that of women, while women have larger neck circumference after menopause compared to women before menopause^24^. Our results showed that the average duration of apnea (P < 0.001) and maximum duration of apnea (P < 0.001) were longer in OSA patients compared to those with non-OSA. The higher the oxygen desaturation index, the longer the duration of apnea, the patients are more likely prone to experience apnea, wake up, hypoxemia and increased partial pressure of carbon dioxide during sleep. Chronic nocturnal sleep apnea induced hypoxemia is strongly associated with the development of hypertension and diabetes, and our results also confirm that the incidence of hypertension (P < 0.001) and diabetes (P = 0.002) is higher in OSA patients than in non-OSA patients. The GOAL questionnaire was developed based on the Brazilian population and has a good effect in screening OSA patients. Considering the differences in genetic background and social behavior, this study verifies the application value of the GOAL questionnaire in screening OSA patients in the Chinese population.

Our results show that the GOAL questionnaire is a highly sensitive questionnaire. However, as sensitivity increases, its specificity decreases, when AHI is 5, 15 and 30 events/h, its sensitivity and specificity are 95.95% and 77.53%, 96.95% and 50.00%, 98.36% and 34.80%, respectively. We have statistically compared the AUCs of the four screening questionnaires, and the results show that there is only a difference between the AUC of STOP-Bang and that of GOAL (P < 0.01). There was no difference in AUC between GOAL and No-Apnea (P > 0.05), NoSAS (P > 0.05), No difference in AUC between No-Apnea and NoSAS (P > 0.05), STOP-Bang (P > 0.05), and NoSAS and STOP-Bang (P > 0.05) are not different. The AUC-ROC value of the GOAL questionnaire was the lowest among the four questionnaires, and the diagnostic efficacy was the lowest, However, considering that our research purpose is to screen moderate and severe OSA patients, so that these moderate and severe OSA patients can get a clear diagnosis through the next PSG examination, and then get treatment. Therefore, we will put the sensitivity of the screening questionnaire above the specificity. Among the four kinds of screening questionnaires, the sensitivity of GOAL questionnaire is the highest, the second is the STOP-Bang questionnaire, which is consistent with our research purpose. GOAL questionnaire is a more sensitive questionnaire, which further reduces the false negative rate of screening OSA patients. Some studies have shown that the neck circumference of false negative participants in the NoSAS score are low, which may be because the sleep breathing disorders of these patients are more likely to be related to maxillofacial malformation, arousal threshold or upper respiratory tract muscle control dysfunction, but not related to obesity, which still needs further research^14,27^.

In this study, different AHI cut-off values were used to diagnose or exclude patients with OSA. In the sleep laboratory, our main goal is to identify patients with severe OSA who require CPAP therapy, in which case we should prefer a higher AHI cut-off value, but in primary health-care centers, we prefer a lower AHI cut-off value considering not missing cases of OSA patients.

## Strengths and limitations

The strength of this study was the fact that it analyzed the application value of four screening tools in sleep laboratory patients. This study has some limitations. The study population was selected from a sleep laboratory, which itself has a high prevalence of OSA, and the predictive value was influenced by prevalence with some selection bias. Second, the total number of cases in the study was small, so this study needs to be validated in other sleep centers and in a larger number of other populations; in addition, male patients made up a larger proportion of our study population, which may have influenced the results.

## Conclusion

The GOAL questionnaire has good predictive value for OSA, which is a clinically applicable questionnaire with similar performance to the No-Apnea score, STOP-Bang questionnaire and NoSAS score. As with any demographic study, more studies with diverse populations are needed to validate the predictive performance of OSA screening tools.

## Supplementary Information

Supplementary Tables.

## Data Availability

All data generated or analyzed during this study are included in this published article (and its supplementary information files).
