# Supplementary material for: Utilization of the GOAL questionnaire as a standardized screening tool for obstructive sleep apnea
Source: Sci Rep. 2023 Apr 4;13:5489. doi: 10.1038/s41598-023-31247-x (PMC10073216; doi:10.1038/s41598-023-31247-x)
Supplement: Supplementary file 1 — Supplementary Tables. [file 41598_2023_31247_MOESM1_ESM.docx]

# Appendices

#

**OSA Screening Questionnaire**

Table S1 Stop-Bang Questionnaire

| Question | Reply | Reply |
| --- | --- | --- |
| S Snoring： Are you snoring loudly (bigger than talking, or loud enough to be heard with the door closed)? | Yes | No |
| T Tired： Do you often feel tired, sleepy or lethargic during the day | Yes | No |
| O Observed： Has anyone noticed that your breathing stops while you sleep? | Yes | No |
| P Blood pressure： Do you have high blood pressure? Or are you being treated for high blood pressure? | Yes | No |
| B BMI：>35kg/m2 | Yes | No |
| A Age：>50 years | Yes | No |
| N Neck circumference：>40cm | Yes | No |
| G Gender: Male? | Yes | No |

Table S2 NoSAS score

| Project | Result |
| --- | --- |
| 1. Neck circumference (cm) | <=40  ＞40 |
| 2.BMI (kg/m2) | <25  25-29.99  >=30 |
| 3. Snoring | No  Yes |
| 4. Age (years) | <=55  >55 |
| 5. Gender | Male  Female |

Table S3 No-apnea score

| Project | Result |
| --- | --- |
| 1.Neck circumference (cm) | 37-39.9cm (1score)  40-42.9cm (3score)  ≥43cm (6 score) |
| 2. Age | 35-44 years（1 score）  45-54 years（2 score  ≥55 years （3 score） |

Table S4 GOAL questionnaire

| Project | Result |
| --- | --- |
| Male | Yes：1 score No： 0 score |
| BMI≥30 | Yes：1 score No： 0 score |
| Age≥50 | Yes：1 score No：0 score |
| loud snoring | Yes：1 score No：0 score |
